# S245. LOWER- AND HIGHER-LEVEL SOCIAL COGNITIVE FACTORS ACROSS INDIVIDUALS WITH SCHIZOPHRENIA SPECTRUM DISORDERS AND HEALTHY CONTROLS: RELATIONSHIP WITH NEUROCOGNITION AND FUNCTIONAL OUTCOME

**DOI:** 10.1093/schbul/sby018.1032

**Published:** 2018-04-01

**Authors:** Lindsay Oliver, John Haltigan, James Gold, George Foussias, Pamela DeRosse, Robert Buchanan, Anil Malhotra, Aristotle Voineskos

**Affiliations:** 1 Centre for Addiction and Mental Health; 2 Maryland Psychiatric Research Center; 3 Zucker Hillside Hospital

## Abstract

**Background:**

Individuals with schizophrenia spectrum disorders (SSDs) often suffer social cognitive deficits, which are associated with functional outcome. These include lower-level “simulation” processes (emotion recognition), thought to be subserved by a frontoparietal circuit, and higher-level “mentalizing” processes (theory of mind), involving cortical midline and lateral temporal regions. Despite evidence supporting the distinction of these constructs, little work has focused on the factor structure of social cognition. In schizophrenia, factor analytic results have been inconsistent, likely due to task and analytic approach variability, and inadequate sample sizes. Further, confirmatory factor analysis (CFA) has not been used to compare multiple models across people with SSDs and healthy controls. Thus, our objective was to elucidate the factor structure of social cognition across a large group of people with SSDs and healthy controls. We hypothesized that a two-factor model, including simulation and mentalizing factors, would demonstrate the best fit across participants. We also expected social cognitive and neurocognitive factors to load on separate respective higher-order factors, and social cognition to mediate the relationship between neurocognition and clinical and functional outcome measures.

**Methods:**

Behavioural data was collected from 164 participants with SSDs and 102 healthy controls across three sites. Participants completed four tasks including measures of social cognition, ranging from basic emotion recognition to complex mental state inference. Participants also completed measures of functional outcome, symptom ratings, and the MATRICS Consensus Cognitive Battery. CFAs were conducted to test social cognitive models, as well as models of social cognition and neurocognition, and multi-group CFA was used to test measurement invariance between patients and controls.

**Results:**

As predicted, a two-factor (simulation, mentalizing) model fit the social cognitive data well across participants with SSDs and healthy controls (RMSEA = .010, CFI = 1.00). This model also fit significantly better than a one-factor model (p < .001). Further, measurement invariance testing revealed factor structure invariance, loading invariance, and partial intercept invariance between groups, allowing for between-group comparisons. Participants with SSDs showed lower scores than controls for both simulation and mentalizing factors (p < .001), and scores on both factors correlated significantly with symptom ratings and functional outcome measures. Including neurocognitive data, a higher-order two-factor (social cognition, neurocognition) model fit the data well (RMSEA = .047, CFI = .971), and showed significantly better fit than a one- or two-factor model (p < .001). Lastly, social cognition was found to mediate the relationship between neurocognition and negative symptoms, as well as social functioning and quality of life measures (p < .05).

**Discussion:**

Our results provide evidence that social cognition includes lower- and higher-level dimensions across both individuals with SSDs and healthy controls. They also suggest that both aspects are associated with clinical and functional outcome indices, and act as a mediator between neurocognition and these measures. This provides support for distinguishing lower- and higher-level social cognition between and across people with SSDs and healthy controls, and suggests that they may indeed have partially distinct underlying mechanisms. Further, results confirm the importance of social cognition as it relates to clinical and functional outcomes, and thereby as a potential treatment target for patients with SSDs.

